# The spinal anti-inflammatory mechanism of motor cortex stimulation: cause of success and refractoriness in neuropathic pain?

**DOI:** 10.1186/s12974-014-0216-1

**Published:** 2015-01-20

**Authors:** Guilherme D Silva, Patrícia SS Lopes, Erich T Fonoff, Rosana L Pagano

**Affiliations:** Laboratory of Neuromodulation and Experimental Pain, Hospital Sírio Libanês, Rua Coronel Nicolau dos Santos, 69, 01308-060 São Paulo, SP Brazil; Division of Functional Neurosurgery, Department of Neurology, University of São Paulo School of Medicine, Rua Dr Ovídio Pires de Campos, 785, 01060-970 São Paulo, SP Brazil

**Keywords:** Motor cortex, Epidural stimulation, Neuropathic pain, Glia, Cannabinoids, Neuroinflammation, Spinal cord, Rats

## Abstract

**Background:**

Motor cortex stimulation (MCS) is an effective treatment in neuropathic pain refractory to pharmacological management. However, analgesia is not satisfactorily obtained in one third of patients. Given the importance of understanding the mechanisms to overcome therapeutic limitations, we addressed the question: what mechanisms can explain both MCS effectiveness and refractoriness? Considering the crucial role of spinal neuroimmune activation in neuropathic pain pathophysiology, we hypothesized that modulation of spinal astrocyte and microglia activity is one of the mechanisms of action of MCS.

**Methods:**

Rats with peripheral neuropathy (chronic nerve injury model) underwent MCS and were evaluated with a nociceptive test. Following the test, these animals were divided into two groups: MCS-responsive and MCS-refractory. We also evaluated a group of neuropathic rats not stimulated and a group of sham-operated rats. Some assays included rats with peripheral neuropathy that were treated with AM251 (a cannabinoid antagonist/inverse agonist) or saline before MCS. Finally, we performed immunohistochemical analyses of glial cells (microglia and astrocytes), cytokines (TNF-α and IL-1β), cannabinoid type 2 (CB2), μ-opioid (MOR), and purinergic P2X4 receptors in the dorsal horn of the spinal cord (DHSC).

**Findings:**

MCS reversed mechanical hyperalgesia, inhibited astrocyte and microglial activity, decreased proinflammatory cytokine staining, enhanced CB2 staining, and downregulated P2X4 receptors in the DHSC ipsilateral to sciatic injury. Spinal MOR staining was also inhibited upon MCS. Pre-treatment with AM251 blocked the effects of MCS, including the inhibitory mechanism on cells. Finally, MCS-refractory animals showed similar CB2, but higher P2X4 and MOR staining intensity in the DHSC in comparison to MCS-responsive rats.

**Conclusions:**

These results indicate that MCS induces analgesia through a spinal anti-neuroinflammatory effect and the activation of the cannabinoid and opioid systems via descending inhibitory pathways. As a possible explanation for MCS refractoriness, we propose that CB2 activation is compromised, leading to cannabinoid resistance and consequently to the perpetuation of neuroinflammation and opioid inefficacy.

**Electronic supplementary material:**

The online version of this article (doi:10.1186/s12974-014-0216-1) contains supplementary material, which is available to authorized users.

## Background

Neuropathic pain is defined as pain caused by a primary lesion of the somatosensory system [1]. Classic symptoms include allodynia (pain in response to innocuous thermal or mechanical stimuli), hyperalgesia (exaggerated pain in response to noxious stimuli), and spontaneous pain (pain in the absence of noxious stimulation) [2-4]. These symptoms can be explained by immunogenic and neurogenic mechanisms such as glial activation, proinflammatory cytokine release, and differential activation of receptors [3,5,6]. Proinflammatory cytokines, such as IL-1β and TNF-α, are secreted by activated astrocytes and microglia, enhancing glutamatergic transmission and disinhibiting GABAergic interneurons in the dorsal horn of the spinal cord (DHSC). Through this process, spinal synaptic transmission is increased, leading to neuropathic pain sensitization [7-9].

The cannabinoid and purinergic systems modulate the inflammatory response of glial cells. Cannabinoid type 2 receptor (CB2) activation in glial cells decreases IL-1β and TNF-α release [7,10,11], while the binding of adenosine triphosphate (ATP) to purinergic P2X4 receptors in microglia leads to the secretion of inflammatory mediators [12].

Epidural motor cortex stimulation (MCS) has been proposed as a treatment for neuropathic pain that is refractory to clinical management (i.e. tricyclic antidepressants, antiepileptic drugs, or opioids) [13-18]. Moreover, MCS is a nondestructive, adjustable, and reversible therapy for central and peripheral pain [19]. Although MCS is considered a promising strategy for drug-resistant neuropathic pain, one third of patients do not respond satisfactorily [20], and its mechanism of action remains controversial. In rats and humans, both the opioid system and descending analgesic pathways have been implicated in MCS-induced analgesia [21-27]. Even though neuroimmunomodulation processes have been implicated in the pathophysiology of neuropathic pain, their underlying mechanisms in MCS-induced analgesia remain poorly understood. In this study, using an experimental model of peripheral neuropathy, we investigated whether modulation of spinal glial inflammatory response is involved in the mechanisms of MCS-induced analgesia. We also studied whether the differential involvement of cannabinoid, opioid, and purinergic systems plays a role in the effectiveness and refractoriness to MCS.

## Methods

### Experimental design

Under anesthesia, peripheral neuropathy (or sham surgery) was performed on the right hind limb of adult rats (day 0). After 1 week, transdural electrodes were implanted over the left motor cortex involving the functional area of the right hind limb (day 7). After another week, the nociceptive test (described in the *Measuring mechanical hyperalgesia* section below) was performed in awake animals (day 14). After that, a group of rats with peripheral neuropathy was submitted to 15 minutes of MCS and, at the end of this period, still under stimulation, they were re-evaluated on the nociceptive test. Sham-operated and neuropathic rats that were not stimulated were also evaluated. To evaluate the cannabinoid involvement, animals with peripheral neuropathy were treated with cannabinoid receptor antagonist or saline, and after 1 hour, they were stimulated and re-evaluated with the nociceptive test. Following these experiments, animals were divided into eight groups: sham-operated, CCI (non-stimulated), CCI + MCS (MCS-responsive), and CCI + MCS (MCS-refractory); or CCI + Saline, CCI + Saline + MCS, CCI + AM251 + MCS, and Naive + AM251. Immediately after the last nociceptive evaluation, all animals were anesthetized, perfused, and their spinal cords were processed for immunohistochemical analysis.

### Animals

Male Wistar rats weighing between 180 and 220 g were housed in acrylic boxes (3 rats per box) for at least 2 days before initiating experimental procedures. Animals were maintained in a controlled environment under 12/12 hour light/dark cycle at room temperature (22° ± 2°C) with wood shavings and free access to water and rat chow pellets. The experimental procedures were in accordance with the guidelines for the ethical use of animals in research involving pain and nociception [28], and were approved by the Ethics Committee on the Use of Animals at Hospital Sírio Libanês (São Paulo, Brazil), under protocol number CEUA 2012/24.

### Neuropathic pain induction

Rats received an inhalational general anesthetic (halothane, 2.5%) and were subjected to sciatic nerve chronic constriction injury (CCI), according to previously described methods [29]. Using sharp and blunt dissection, the sciatic nerve of the right paw was exposed at the midline of the thigh. Proximal to the sciatic nerve’s trifurcation (about 7 mm), 4 loose ligatures were placed around the nerve using 4.0 catgut chrome wire. The skin was closed with 4.0 nylon. In sham-operated rats, the sciatic nerve was exposed but not compressed.

### Electrode implantation and electrical stimulation parameters

One week after the induction of peripheral neuropathy or sham surgery, rats received local (2% lidocaine 100 μL, subcutaneously) and general (ketamine/xylazine 0.5/2.3 mg/kg, intramuscularly) anesthesia. Whenever necessary, supplementary doses of ketamine were administered to the animals. Guided by a map developed by our group [30], two transdural stainless steel electrodes were placed under stereotaxic conditions on the primary motor cortex over the functional area of the hind limb. Two fixation screws and acrylic polymer were used to stabilize the implant and to ensure electrical isolation. One week after surgical implantation, electrical stimulation was delivered in a single 15-minute session with the following parameters: 60 Hz, 1.0 V, and 210 μs (Medtronic electrical stimulator, Minneapolis, MN, USA), which has been previously shown to reverse the neuropathic pain in rats [31].

### Pharmacological treatment

Fourteen days after CCI, animals were treated with AM251, a cannabinoid receptor antagonist/inverse agonist (1 mg/kg, intraperitoneally; Sigma-Aldrich, St Louis, MO, USA) [32] or with saline solution (400 μL, 0.9% NaCl in water) 1 hour before the nociceptive test or MCS. Naive animals injected with AM251 were also evaluated. The experimenter was blind to the treatments.

### Measuring mechanical hyperalgesia

The mechanical nociceptive threshold was determined using a pressure apparatus on the right hind paw (Insight Ltda., Ribeirão Preto, São Paulo, Brazil), as previously described [33]. Briefly, the mass (in grams) required to induce a withdrawal response represented the nociceptive threshold. The nociceptive test was carried out on the 14th day following CCI or sham surgery, before (initial measurement) and during MCS (final measurement). Upon antagonist treatment, the nociceptive threshold was evaluated before CCI (initial measurement), 14 days after CCI (final measurement), and 1 hour after antagonist or saline administration during MCS (final measurement + MCS). The results were analyzed by comparing the initial and final measurements to each other. Investigators were blind to group identification. To reduce stress, rats were habituated to the testing procedure the day preceding the experiment. According to the presence or absence of analgesia (as measured by significant changes in nociceptive threshold), the MCS group was further divided into ‘MCS-responsive’ and ‘MCS-refractory’ groups.

### Immunohistochemistry

Immediately after the last nociceptive test, rats were deeply anesthetized with ketamine and xylazine and then subjected to transcardiac perfusion with saline solution, followed by 4% paraformaldehyde (PFA) dissolved in 0.1 M phosphate buffer (PB). The lumbar spinal cords (L2-L5 segments) were collected and post-fixed in PFA for 4 hours, followed by incubation with 30% sucrose solution for 48 hours at 4°C. Coronal sections (30 μm) were cut on a freezing microtome. Heat-induced epitope retrieval (HIER) in citrate buffer (10 mM) was performed at 75°C for 30 minutes. Next, tissue sections were incubated for 48 hours at 4°C, with the following primary antibodies: mouse anti-GFAP (glial fibrillary acidic protein,1:1,000, G3893, Sigma-Aldrich, St Louis, MO, USA), rabbit anti-Iba-1 (1:1,000, 019-19741, Wako Chemicals, Richmond, VA, USA), goat anti-IL-1β (1:500, AF-501-NA, R & D Systems, Minneapolis, MN, USA), rabbit anti-TNF-α (1:500, AB1837P, Calbiochem, San Diego, CA, USA), rabbit anti-CB2 (1:500, 101550, Cayman, Ann Arbor, USA), goat anti-MOR-1 (μ-opioid receptor 1, 1:500, sc-7488, Santa Cruz Biotechnology, Santa Cruz, CA, USA), and rabbit anti-P2X4 (1:500, APR-002, Alomone Labs, Jerusalem, Israel) diluted in 0.3% of Triton X-100 containing 5% normal donkey serum. Then, sections were incubated for 2 hours at room temperature with biotinylated secondary antibodies (1:200, Jackson ImmunoResearch, Bar Harbor, ME, USA) or fluorescent secondary antibodies: fluorescein isothiocyanate (FITC) (green) or tetramethylrhodamine (TRITC) (red) (1:100, Jackson ImmunoResearch, Bar Harbor, ME, USA). Sections with biotinylated antibodies were incubated for 2 hours at room temperature, with avidin-biotin complex (1:100, ABC Elite kit, Vector Labs, Burlingame, CA, USA), and visualized with 0.05% diaminobenzidine tetrahydrochloride (DAB, Sigma-Aldrich, St Louis, MO, USA) and 0.03% (final concentration) hydrogen peroxide in PB. Then, sections were mounted on glass slides, air-dried, dehydrated, and coverslipped with Permount (Fisher Scientific, Pittsburgh, PA, USA). Sections with fluorescent antibodies were mounted on slides and coverslipped with mounting medium. For both assays, samples were washed between each step (3 × 5 minutes). Finally, images were captured utilizing a fluorescence/light microscope (E1000, Nikon, Melville, NY, USA) and Zen software (Carl Zeiss, Oberkochen, Germany).An area encompassing the DHSC (L4-L5 dorsal horn, ipsilateral to CCI) was outlined and immunoreactivity intensities above background were semiquantified using ImageJ software (National Institutes of Health; http://rsbweb.nih.gov/ij/). For each assay, total number of positive profile (immunostained particles), including neuropil and cell bodies, was used to provide a mean fluorescence value (compared with pre-defined threshold) in five tissue sections per animal and four to six animals per group.

### Statistical analysis

Results are expressed as means ± standard error of the mean (SEM). Data were analyzed with GraphPad Prism (La Jolla, CA, USA) statistical software using ANOVA (analysis of variance) followed by Tukey *post hoc* tests. Three or four groups were compared: sham-operated, CCI (non-stimulated), CCI + MCS (MCS-responsive), and CCI + MCS (MCS-refractory); or CCI + Saline, CCI + Saline + MCS, CCI + AM251 + MCS, and Naive + AM251. In all cases, *P* < 0.05 was considered statistically significant.

## Results and discussion

### Does MCS modulate proinflammatory cytokine release and spinal glial cell activation?

**Figure 1:**
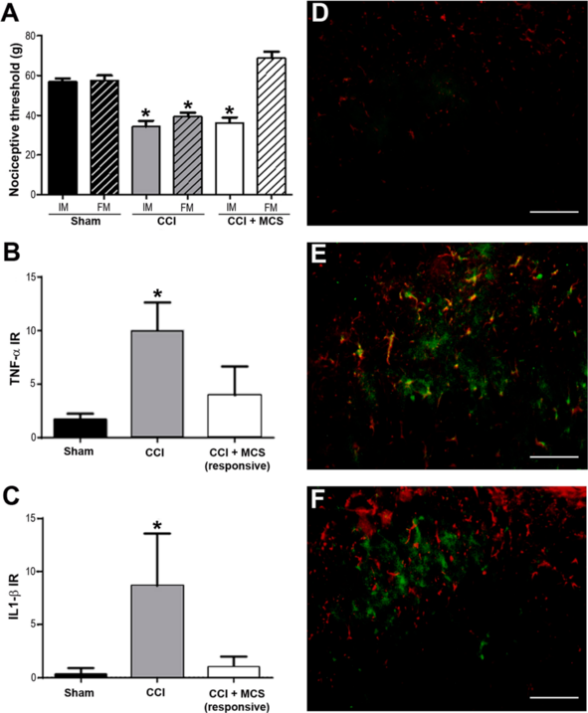
**Motor cortex stimulation (MCS), analgesia, and their relationship with spinal cytokine release. (A)** Nociceptive threshold evaluated in the right hind paw of sham-operated rats (Sham), animals with sciatic nerve chronic constriction injury (CCI), and animals with CCI submitted to MCS (CCI + MCS). CCI was performed in the right paw and cortical electrodes were implanted in the left hemisphere. The nociceptive test was performed 14 days after sham surgery or CCI (IM, initial measurement), and again after 15 minutes, regardless of whether MCS was performed or not (FM, final measurement). Quantification of TNF-α **(B)** and IL-1β **(C)** immunofluorescence-staining in the dorsal horn of the spinal cord (DHSC), ipsilateral to sham or CCI (n = 5 images per animal). Values represent means ± SEM (n = 5 animals per group). *P < 0.05, compared to the sham group. Photomicrographs illustrating glial fibrillary acidic proten (GFAP) (red), IL-1β (green), GFAP and IL-1β colocalization (yellow) in the DHSC of sham **(D)**, CCI **(E)**, and CCI + MCS (responsive) **(F)** animals. Scale bars: 130 μm.

Similar to results previously reported [31], CCI caused mechanical hyperalgesia and MCS reversed this response in 88% of cases (Figure 1A). Three animals (12%) that did not respond to MCS were analyzed separately as the CCI + MCS (MCS-refractory) group. We found no difference between the CCI + MCS (MCS-responsive) and CCI + MCS (MCS-refractory) groups on the initial measure of hyperalgesia (prior to MCS).

In an attempt to elucidate the modulation of glial cell activity in MCS-induced analgesia, we evaluated Iba-1 (microglial marker) and GFAP (astrocytic marker) immunolabeling in the DHSC ipsilateral to the CCI. We observed a marked increase in the density of Iba-1 and GFAP-positive cells in the DHSC of CCI rats in comparison to sham-operated rats. MCS reversed both Iba1 and GFAP increased reactivity in neuropathic rats (Additional file 1), suggesting an association between MCS and changes in activation pattern of the spinal cord astrocytes and microglia. Furthermore, we observed both astrocyte hyperplasia (increased number of cells) and hypertrophy (increased cell volume), in the DHSC of the CCI group as compared with the sham-operated group, consistent with astrocyte activation [34,35]. In morphological analysis, MCS reversed the astrocyte hypertrophy but not hyperplasia. Microglial hyperplasia was clear in CCI compared to sham-operated, characterizing microglial activation [36]. However, the effect of MCS on hyperplasic and hypertrophic responses of spinal microglia, as compared with the CCI group, was not easily detected in our results. To solve this problem, we used P2X4 staining as a marker of microglial activation and the results are discussed below. Considering that glial proinflammatory cytokines mediate the initiation and maintenance of neuropathic pain in animal models [37,38], we evaluated the presence of TNF-α and IL-1β in the DHSC. Fluorescence microscopy analysis revealed that TNF-α (Figure 1B and Additional file 2B) and IL-1β (Figure 1C and Additional file 2E) immunoreactivity was more intense in the CCI group as compared with the sham-operated group (Figure 1B and C, and Additional file 2). Increased cytokine immunoreactivity was completely reversed by MCS (Figure 1B and C, and Additional file 2). These findings are consistent with the observed analgesic effects, suggesting a correlation between MCS and downregulation of central sensitization mechanisms, as cytokines levels and glia (astrocytes and microglia) activation patterns. We hypothesize that, similar to the mechanism associated with drugs (xanthine derivative KMUP-1, fucoidan, montelukast) [39-41], hyperalgesia in neuropathic pain is attenuated upon suppression of spinal neuroinflammation. To the best of our knowledge, this is the first study to show the spinal anti-inflammatory effect of MCS.

Accumulating evidence has shown that activated astrocytes contribute to maintaining chronic pain sensitization through IL-1β release in the spinal cord under peripheral nerve injury [42-44]. In agreement with these findings, we observed colocalization of IL-1β and astrocytes (GFAP) in the CCI group (Figure 1E), but not in the CCI + MCS (MCS-responsive) (Figure 1F) or sham-operated animals (Figure 1D). Thus, we propose that the anti-neuroinflammatory effects of MCS depend on downregulation of glial cell activity.

### Can modulatory systems (cannabinoid, opioid, and purinergic) explain the positive effect or refractoriness of MCS?

**Figure 2:**
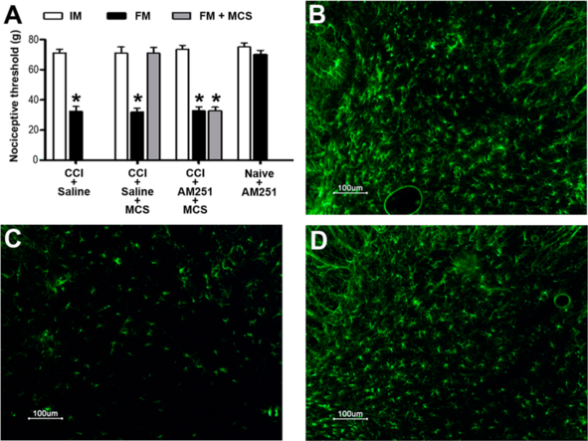
**Participation of the cannabinoid system in motor cortex stimulation (MCS)-induced analgesia. (A)** Nociceptive threshold evaluated in the right hind paw of rats with peripheral neuropathy pre-treated with saline (CCI + saline), chronic constriction injury (CCI) rats pre-treated with saline and submitted to MCS (CCI + Saline + MCS), and CCI rats pre-treated with cannabinoid receptor antagonist AM251 and submitted to MCS (CCI + AM251 + MCS). CCI was performed in the right paw and cortical electrodes were implanted in the left hemisphere. The nociceptive test was performed before CCI (IM, initial measurement), and 14 days after CCI (FM, final measurement). One hour after injection of saline (400 μL) or AM251 (1 mg/kg; intraperitoneally), animals were submitted or not to MCS and re-evaluated on the nociceptive test (FM + MCS). Naive rats pre-treated only with AM251 were also evaluated. Values represent the mean ± SEM (n = 5 per group). *P < 0.05 compared with IM. Photomicrographs illustrating glial fibrillary acidic protein (GFAP) immunofluorescence in the dorsal horn of the spinal cord (DHSC) of CCI **(B)**, CCI + Saline + MCS **(C)**, and CCI + AM251 + MCS **(D)** animals.

Endocannabinoids inhibit the neuroinflammatory response [45], and cannabinoid receptor agonists have been shown to have beneficial effects in several animal models of neuropathic pain [46,47]. In an attempt to evaluate the participation of the endocannabinoid system in MCS-induced analgesia, animals were treated with the cannabinoid receptor antagonist/inverse agonist AM251 prior to cortical stimulation. We observed that the analgesic effect of MCS was completely inhibited in animals pre-treated with AM251 (Figure 2A). To verify the role of glia in this response, we evaluated GFAP-positive cells in CCI, CCI + saline + MCS, and CCI + AM251 + MCS groups. As demonstrated by the increased GFAP staining, astrocytes remained activated after pre-treatment with AM251 (Figure 2B-D), suggesting that the endocannabinoid system is involved in MCS-induced analgesia by inhibiting astrocyte activity. Given that (1) MCS inhibits astrocyte and microglial activation; (2) the CB2 receptor, rather than CB1, is directly involved in astrocyte and microglial inhibition in neuropathic pain conditions [47-49]; and (3) AM251 can act as a CB2 inverse agonist [50,51], we next evaluated the CB2 labeling pattern in the spinal cord of MCS-responsive and MCS-refractory animals.

While the CB2 receptor is the main receptor for cannabinoid signaling in astrocytes and microglia, the MOR is found exclusively in DHSC neurons [11,52-54]. While the former decreases cytokine release (IL-1β and TNF-α) from astrocytes and microglia [55,56], the latter diminishes neurotransmitter secretion (glutamate, substance P) in the DHSC upon activation of the descending analgesic pathway [57,58]. Because both mechanisms play a crucial role in neuropathic pain pathophysiology, the cannabinoid antagonist/inverse agonist AM251 as well as the opioid antagonist naloxone [24] can compromise MCS-induced analgesia.

**Figure 3:**
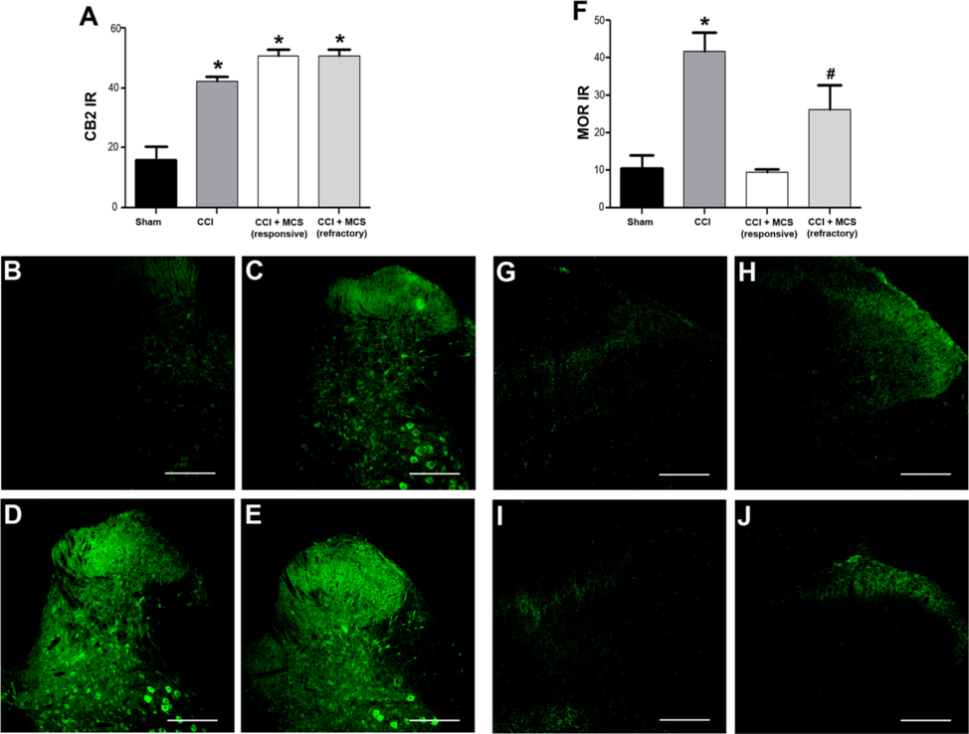
**Spinal cannabinoid receptor type 2 (CB2) and μ-opioid receptor (MOR) staining and its correlation with the effectiveness of motor cortical stimulation (MCS).** Quantification of CB2 **(A)** and MOR **(F)** immufluorescence-staining in the dorsal horn of the spinal cord (DHSC), ipsilateral to sham surgery or chronic constriction injury (CCI), in sham-operated rats (Sham), CCI rats with neuropathic pain (CCI), CCI rats in which MCS reversed the neuropathic pain (CCI + MCS, responsive), and CCI rats unresponsive to MCS (CCI + MCS, refractory). Values represent the mean ± SEM (n = 5 images per animal, n = 3 to 5 animals per group). *P < 0.05 compared to the sham group. Photomicrographs illustrating CB2 **(B-E)** and MOR **(G-J)** immunofluorescence in the DHSC, ipsilateral to sham surgery or CCI, in sham **(B, G)**, CCI **(C, H)**, CCI + MCS (responsive) **(** **D, I** **)**, and CCI + MCS (refractory) **(E, J)** animals. Scale bars: 130 μm.

Cannabinoid and opioid receptor regulation work in opposite directions: while CB2 operates in a positive feedback loop in which receptor activation leads to increased receptor expression, MOR is downregulated with increased opioid transmission, and its internalization correlates with the MOR-mediated postsynaptic inhibitory effect [11,59]. Given that microglial cells are activated [48,49,52] and spinal opioid neurotransmitters are depleted during chronic pain [60,61], we observed, as expected, a more intense staining for both spinal CB2 (Figure 3A, C) and MOR (Figure 3F, H) receptors in rats with peripheral neuropathy in comparison to sham-operated rats (Figure 3A, B, F, G). Also, MCS further increased CB2 (Figure 3A, D) and decreased MOR reactivity (Figure 3F, I) in MCS-responsive animals, results that are coherent with the activation of opioid and cannabinoid systems and their roles in the reversal of neuropathic pain [62-65]. Unexpectedly, no difference in spinal CB2 immunoreactivity was observed between MCS-responsive and MCS-refractory groups (Figure 3A, E). On the other hand, MOR staining in unresponsive rats was partially inhibited when compared with responsive and sham-stimulated rats (Figure 3F, J). Considering that (1) the inflammatory state modulates opioid release in neuropathic pain conditions [66]; and (2) astrocytes and microglia remained activated in MCS-refractory animals, a plausible explanation for the partial reversion of MOR upregulation in this group is that inflammatory mediators secreted by activated astrocytes and microglia inhibited opioid release of opioid peptides.

**Figure 4:**
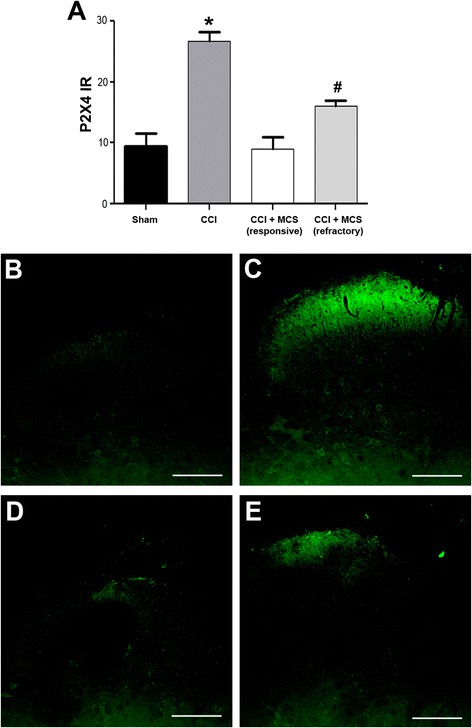
**Spinal purinergic system involvement in analgesia induced by motor cortex stimulation (MCS). (A)** Quantification of P2X4 immufluorescence-staining in the dorsal horn of the spinal cord (DHSC), ipsilateral to sham surgery or chronic constriction injury (CCI), in sham-operated rats (Sham), CCI rats with neuropathic pain (CCI), CCI rats in which MCS reversed the neuropathic pain (CCI + MCS, responsive), and CCI rats unresponsive to MCS (CCI + MCS, refractory). Values represent means ± SEM (n = 5 images per animal, n = 3 to 5 animals per group). *P < 0.05 compared to the sham group. #*P* < 0.05 compared to the sham and CCI groups. Photomicrographs illustrating P2X4 immunofluorescence in the DHSC, ipsilateral to sham surgery or CCI, in sham **(B)**, CCI **(C)**, CCI + MCS (responsive) **(D)**, and CCI + MCS (refractory) **(E)** animals. Scale bars: 130 μm.

To better understand these results, we evaluated the purinergic receptor P2X4, which is expressed in activated microglia but not in neurons or astrocytes [67,68]. Animals with peripheral neuropathy showed a significant increase in spinal P2X4 reactivity (Figure 4A, C), which was partially reversed in MCS-responsive rats (Figure 4A, D) when compared with sham-operated animals (Figure 4A, B). Surprisingly, P2X4 staining in the CCI + MCS (MCS-refractory) animals fell somewhere between staining in CCI rats and CCI + MCS (MCS-responsive) rats (Figure 4A, E). Thus, even though cannabinoid transmission was similar regardless of the success or failure of MCS treatment, MCS inhibitory effects on astrocytes and microglia were reduced in the MCS-refractory group. We hypothesize that CB2 activation may be compromised in MCS-refractory animals, probably due to altered intracellular signaling that prevents the inhibition of spinal microglia activity, leading to cannabinoid resistance.

**Figure 5:**
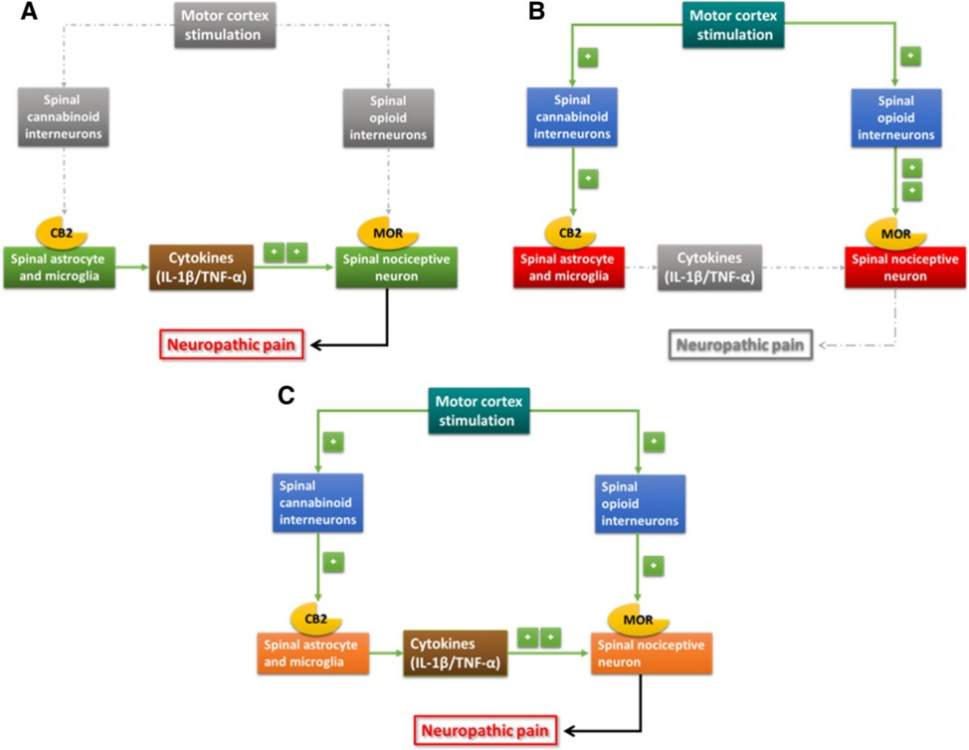
**Motor cortex stimulation (MCS) effectiveness and refractoriness: cannabinoids, opioids, and neuroinflammation. (A)** In the chronic constriction injury (CCI) group, activated astrocytes and microglia release cytokines (IL-1β and TNF-α) that enhance nociceptive neuron transmission in the dorsal horn of the spinal cord (DHSC), resulting in neuropathic pain. **(B)** In CCI + MCS (responsive) group, MCS activates the spinal cannabinoid and opioid interneurons. Endocannabinoids bind to CB2 receptors and inhibit cytokine secretion by glial cells, while endogenous opioids interact with μ-opioid receptors (MOR) receptors and suppress neuronal transmission, thus reverting neuropathic pain. **(C)** In CCI + MCS (refractory) group, endocannabinoids fail to suppress astrocyte and microglia activation (cannabinoid resistance), maintaining proinflammatory cytokine release (perpetuation of neuroinflammation), and thus compromising opioid effectiveness (opioid inefficacy). Hence, spinal pain transmission persists, preventing analgesia.

Cannabinoid resistance is implicated in opioid inefficacy [69], since neuroinflammation persists if cannabinoid modulation fails to suppress astrocyte and microglia activity. Considering this intimate relationship between cannabinoid and opioid systems in DHSC and the fact that MCS activates descending analgesic pathways [21,25,26,70] and inhibits spinal nociceptive neurons [31,71], we propose that the following spinal circuitry is involved in MCS-induced analgesia: activation of spinal cannabinoid neurons causes them to release endocannabinoids, which inhibit astrocyte and microglia activity, thus decreasing neuroexcitatory cytokine secretion and suppressing nociceptive neuron excitability through opioid activation (Figure 5).

A possible explanation for MCS effectiveness in some individuals who are refractory to conventional pharmacological treatments (opioid analgesic, tricyclic antidepressants, and antiepileptic drugs) is that such treatments do not simultaneously modulate cannabinoid and opioid systems in such a manner as to block neuroexcitatory astrocyte and microglia signals and, at the same time, suppress the nociceptive neuron excitability. Future studies should further investigate the mechanisms involved in ‘cannabinoid resistance’: are different receptors involved or are there intracellular signaling abnormalities? Are gene polymorphisms or transcription factor mutations involved?

## Conclusion

Our results indicate that the spinal anti-neuroinflammatory effect of MCS is responsible, at least in part, for the induced analgesia. In addition, the results reinforce that MCS reverses neuropathic pain through the activation of descending analgesic pathways. Our data suggest that through the cannabinoid system, MCS inhibits spinal astrocyte and microglia activity, decreasing proinflammatory cytokine secretion and, thus, neuroinflammation; through the opioid system, MCS suppresses spinal nociceptive neuron excitability and, hence, transmission of persistent pain. The fact that inflammation decreases the efficacy of opioids suggests that both of these mechanisms play a role in MCS-induced analgesia. A possible explanation for MCS refractoriness in some individuals with neuropathic pain is that spinal CB2 activation is compromised, leading to cannabinoid resistance and consequently to the perpetuation of neuroinflammation and opioid inefficacy.

## Additional files

Additional file 1:
**Motor cortex stimulation and spinal glial cells.** Photomicrographs illustrating Iba-1 **(A-C)** and GFAP **(D-F)** staining in the DHSC of sham-operated rat **(A**, **D)**, rat with sciatic nerve chronic constriction injury (CCI) **(B**, **E)**, and rat with CCI submitted to cortical stimulation that was MCS-responsive **(C**, **F)**. CCI was performed in the right paw and cortical electrodes were implanted in the left hemisphere. Sections illustrate the DHSC ipsilateral to sham surgery or CCI. Scale bars: 100 μm **(** **A-C** **)** and 130 μm **(** **D-F** **)**. Additional file 2:
**Motor cortex stimulation and spinal cytokine release.** Photomicrographs illustrating TNF-α **(A-C)** and IL1-β **(D-F)** staining in the DHSC of sham-operated rat **(A**, **D)**, rat with sciatic nerve chronic constriction injury (CCI) **(B**, **E)**, and rat with CCI submitted to cortical stimulation, MCS-responsive **(C**, **F)**. CCI was performed in the right paw and cortical electrodes were implanted in the left hemisphere. Sections illustrate the DHSC ipsilateral to sham surgery or CCI. Scale bars:130 μm.

## Abbreviations

ANOVA: analysis of variance
ATP: adenosine triphosphate
CB2: cannabinoid receptor type 2
CCI: chronic constriction injury
DAB: diaminobenzidine tetrahydrochloride
DHSC: dorsal horn of the spinal cord
FITC: fluorescein isothiocyanate
FM: final measurement
GFAP: glial fibrillary acidic protein
HIER: heat-induced epitope retrieval
IL-1β: interleukin-1β
IM: initial measurement
MCS: motor cortex stimulation
MOR: μ-opioid receptor
PB: phosphate buffer
PFA: paraformaldehyde
TNF-α: tumor necrosis factor-α
TRITC: tetramethylrhodamine
