# Supplementary figures and images for: The spinal anti-inflammatory mechanism of motor cortex stimulation: cause of success and refractoriness in neuropathic pain?
Source: J Neuroinflammation. 2015 Jan 20;12:10. doi: 10.1186/s12974-014-0216-1 (PMC4311417; doi:10.1186/s12974-014-0216-1)

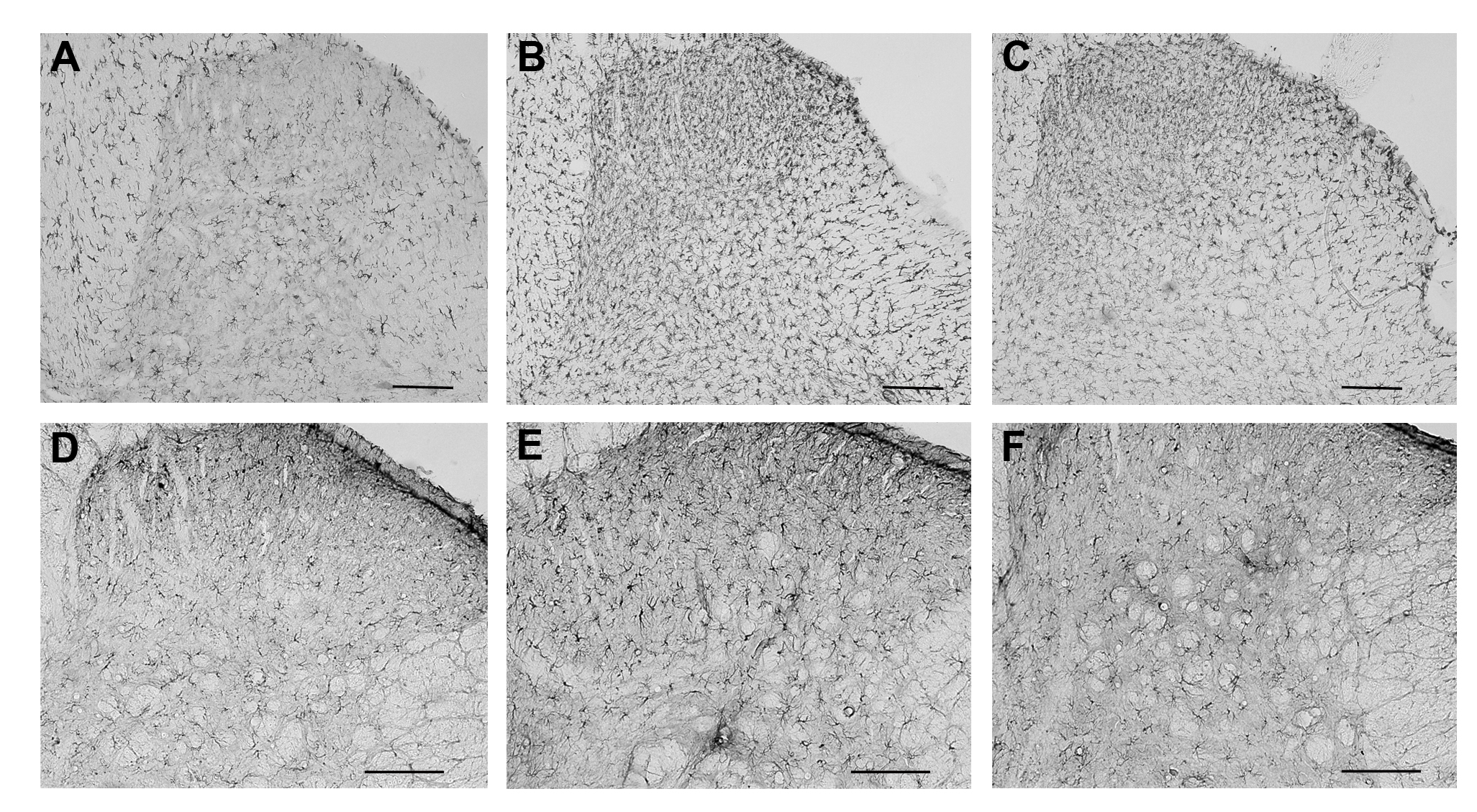

Supplement: Additional file 1: — Motor cortex stimulation and spinal glial cells. Photomicrographs illustrating Iba-1 (A-C) and GFAP (D-F) staining in the DHSC of sham-operated rat (A, D), rat with sciatic nerve chronic constriction injury (CCI) (B, E), and rat with CCI submitted to cortical stimulation that was MCS-responsive (C, F). CCI was performed in the right paw and cortical electrodes were implanted in the left hemisphere. Sections illustrate the DHSC ipsilateral to sham surgery or CCI. Scale bars: 100 μm ( A-C ) and 130 μm ( D-F ). [file 12974_2014_216_MOESM1_ESM.jpeg]

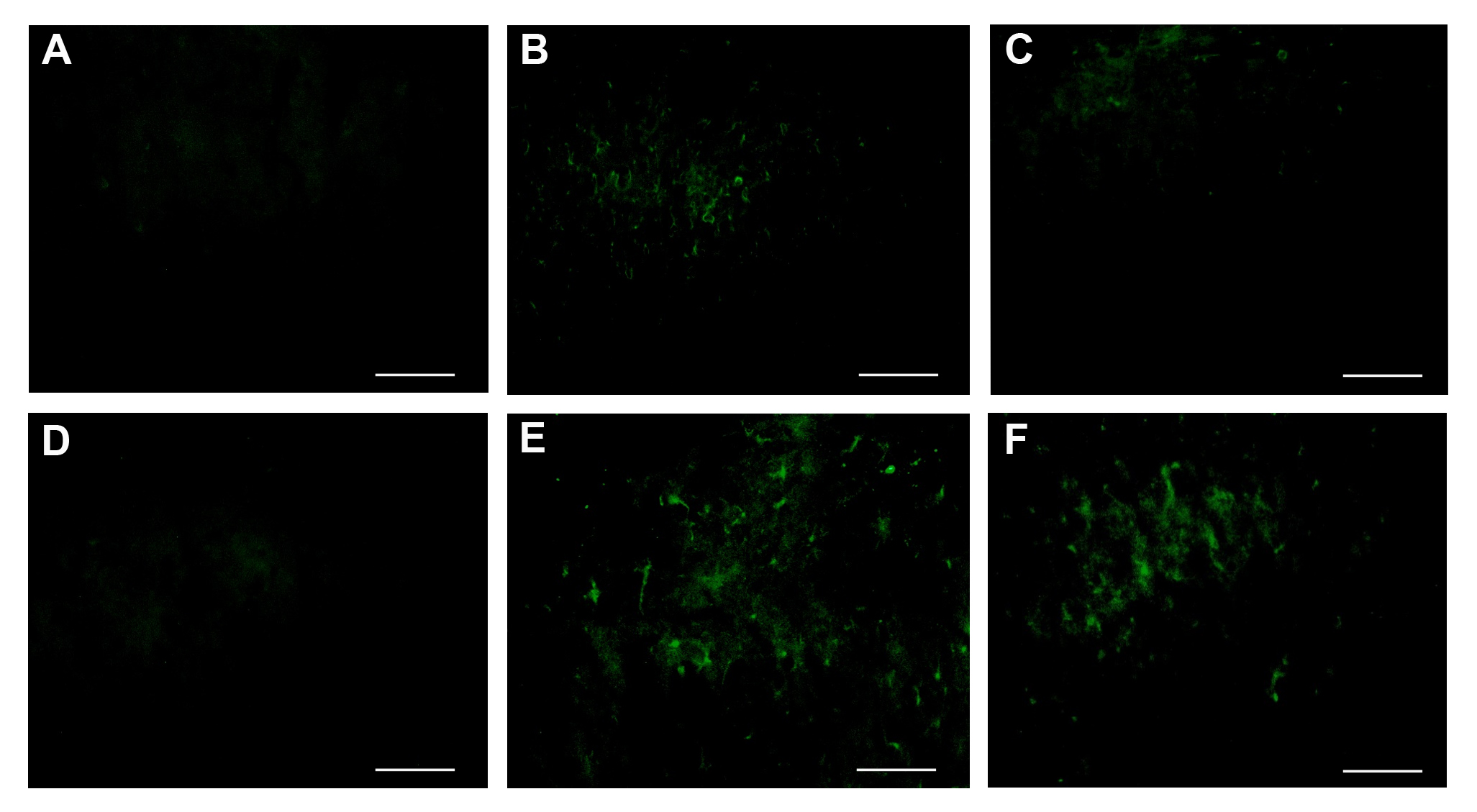

Supplement: Additional file 2: — Motor cortex stimulation and spinal cytokine release. Photomicrographs illustrating TNF-α (A-C) and IL1-β (D-F) staining in the DHSC of sham-operated rat (A, D), rat with sciatic nerve chronic constriction injury (CCI) (B, E), and rat with CCI submitted to cortical stimulation, MCS-responsive (C, F). CCI was performed in the right paw and cortical electrodes were implanted in the left hemisphere. Sections illustrate the DHSC ipsilateral to sham surgery or CCI. Scale bars:130 μm. [file 12974_2014_216_MOESM2_ESM.jpeg]
